# Naive Bayes classifiers for verbal autopsies: comparison to physician-based classification for 21,000 child and adult deaths

**DOI:** 10.1186/s12916-015-0521-2

**Published:** 2015-11-25

**Authors:** Pierre Miasnikof, Vasily Giannakeas, Mireille Gomes, Lukasz Aleksandrowicz, Alexander Y. Shestopaloff, Dewan Alam, Stephen Tollman, Akram Samarikhalaj, Prabhat Jha

**Affiliations:** Centre for Global Health Research, St. Michael’s Hospital, Toronto, Ontario Canada; Department of Statistical Sciences, University of Toronto, Toronto, Ontario Canada; Centre for Control of Chronic Diseases, International Centre for Diarrhoeal Diseases Research, Dhaka, Bangladesh; Medical Research Council/Wits University Rural Public Health and Health Transitions Research Unit (Agincourt), School of Public Health, Faculty of Health Sciences, University of the Witwatersrand, Johannesburg, South Africa; Department of Mechanical and Industrial Engineering, Ryerson University, Toronto, Ontario Canada; Dalla Lana School of Public Health, University of Toronto, Toronto, Canada

**Keywords:** Cause of death (COD), Computer-coded verbal autopsy (CCVA), InterVA, Tariff, Naive Bayes classifier, Physician certified verbal autopsy (PCVA), Verbal autopsy (VA)

## Abstract

**Background:**

Verbal autopsies (VA) are increasingly used in low- and middle-income countries where most causes of death (COD) occur at home without medical attention, and home deaths differ substantially from hospital deaths. Hence, there is no plausible “standard” against which VAs for home deaths may be validated. Previous studies have shown contradictory performance of automated methods compared to physician-based classification of CODs. We sought to compare the performance of the classic naive Bayes classifier (NBC) versus existing automated classifiers, using physician-based classification as the reference.

**Methods:**

We compared the performance of NBC, an open-source Tariff Method (OTM), and InterVA-4 on three datasets covering about 21,000 child and adult deaths: the ongoing Million Death Study in India, and health and demographic surveillance sites in Agincourt, South Africa and Matlab, Bangladesh. We applied several training and testing splits of the data to quantify the sensitivity and specificity compared to physician coding for individual CODs and to test the cause-specific mortality fractions at the population level.

**Results:**

The NBC achieved comparable sensitivity (median 0.51, range 0.48-0.58) to OTM (median 0.50, range 0.41-0.51), with InterVA-4 having lower sensitivity (median 0.43, range 0.36-0.47) in all three datasets, across all CODs. Consistency of CODs was comparable for NBC and InterVA-4 but lower for OTM. NBC and OTM achieved better performance when using a local rather than a non-local training dataset. At the population level, NBC scored the highest cause-specific mortality fraction accuracy across the datasets (median 0.88, range 0.87-0.93), followed by InterVA-4 (median 0.66, range 0.62-0.73) and OTM (median 0.57, range 0.42-0.58).

**Conclusions:**

NBC outperforms current similar COD classifiers at the population level. Nevertheless, no current automated classifier adequately replicates physician classification for individual CODs. There is a need for further research on automated classifiers using local training and test data in diverse settings prior to recommending any replacement of physician-based classification of verbal autopsies.

**Electronic supplementary material:**

The online version of this article (doi:10.1186/s12916-015-0521-2) contains supplementary material, which is available to authorized users.

## Background

Most deaths in low- and middle-income countries occur out of hospital and without medical attention and certification at the time of death. Hence, information on causes of death (CODs) is lacking [1]. In these settings, verbal autopsies (VAs), typically involving lay non-medical interviews of living family members or close associates of the deceased about the details of death, with subsequent assignment of COD by a physician, can be used to estimate COD patterns [2, 3]. The quality of a VA depends on whether the respondent lived with the deceased and can recall and convey the key symptoms prior to death. Clinical diagnostic evidence is usually lacking [4].

The validity of VAs has been widely debated [3, 5, 6] but there is no suitable gold standard, as comparisons to hospital datasets are biased by the sharp differences in the age, education or social status of the deceased, and pathogen distribution between medically-unattended and hospital-based deaths [4]. Physician-based classification of CODs has been criticized for being irreproducible and costly (although recent web-based coding has lowered the costs substantially [4]), and these concerns have in part spurred interest in the use of automated assignment of COD from VAs. However, results of comparison studies show conflicting results. One study of 12,000 deaths comparing the Tariff method to hospital-based, diagnostically-confirmed CODs showed that automated methods outperformed physician-classification [7]. Our recent study of 24,000 deaths found that automated methods have poor sensitivity and specificity against the standard of physician classification of individual CODs, with slightly better performance at the population level [8, 9].

Given the relatively low agreement between current automated techniques and physician classification, there is scope to improve the performance of automated methods, by either altering current methods or exploring new ones. The naive Bayes classifier (NBC) is one of the oldest and simplest classification algorithms and has been present in the machine learning literature for some time [10, 11]. Its foundations can be traced back to the work of the Reverend Thomas Bayes in the 18th century [12]. However, despite its simplicity, NBC has been found to yield classification results comparable to more sophisticated techniques [10, 11, 13]. It has also yielded good results in other mortality-classification settings [14, 15]. To our knowledge, there are no tools specifically using NBC to assign COD from VAs.

Here, we introduce NBC for use in VA, and compare its methodology and performance to automated classifiers that use similar methodologies, namely InterVA-4 [16] and an open-source version of the Tariff method [7], on about 21,000 VAs from three countries. The absence of a reasonable gold standard means that such comparisons cannot be a test of validity of the true COD, as physician coding may itself be misclassified. Nevertheless, our goal was to determine if these automated techniques could achieve high agreement, either at the individual or population level, with the widely accepted clinical standard of physician assignment of COD.

## Methods

### Data

We used VA data from the Indian Million Death Study (MDS) [17] and two health and demographic surveillance sites, in Agincourt, South Africa [18], and Matlab, Bangladesh [19]. Table 1 summarizes the features of these datasets, which we have examined previously [2], as well as a subset of a hospital-based study conducted by the Population Health Metrics Research Consortium (PHMRC) [20]. Sample sizes of the MDS, Agincourt, Matlab, and PHMRC datasets, respectively, were 12,255, 5,823, 3,270, and 2,064, and contained deaths at ages 1–59 months, 15–64 years, 20–64 years, and 28 days–11 years. Physician coding guidelines and procedures were similar. The MDS and Agincourt used dual, independent physician coding, while Matlab used single coding followed by a second screening of CODs by a second physician or experienced paramedic [21]. As there were minor differences between studies in their grouping of CODs, we mapped each study to a standardized list of CODs (Additional file 1).

**Table 1:** Description of datasets

Variable	MDS Study	Agincourt Study	Matlab Study	PHMRC Study
Region	India	South Africa	Bangladesh	Multiple
Sample size	12,225	5,823	3,270	2,064
Ages	1-59 months	15-64 years	20-64 years	28 days – 11 years
Number of causes of death	15	16	15	21
Population	Community deaths	Community deaths	Community deaths	Hospital deaths
Cause of death physician classification	Dual, independent coding of VA records, disagreements resolved by reconciliation, and remaining cases by adjudication by a third physician	Dual, independent coding of VA records, disagreements resolved by third physician	Single coding of VA records followed by a second screening by another physician or experienced paramedic	Hospital certified cause of death, including clinical and diagnostic tests

*MDS* Million Death Study, *PHMRC* Population Health Metrics Research Consortium, *VA* verbal autopsy

We applied the NBC, OTM [8] (a minor modification of a previously reported Tariff method [7]), and InterVA-4 [16] classifiers to individual VA records, and compared their assigned CODs to the physician-assigned causes in the studies.

### Training and test sets

To test the performance of NBC and OTM, we randomly selected non-overlapping subsets of 2,300 and 1,000 deaths each for training and testing from the Agincourt and Matlab studies, respectively. InterVA-4 was limited by the number of records available from the MDS; out of the total approximately 12,000 records, 555 were chosen at random and had their narrative sections manually screened for necessary InterVA-4 indicators that were not available in the closed question sections. For consistency, we randomly selected 555 records from each of the Agincourt and Matlab datasets to test the performance of InterVA-4. In the case of the MDS, because of the large amount of available data, we separately examined performance of training on 555 and 11,000 randomly selected records, and testing on a non-overlapping set of 555 records.

Within each dataset, the NBC and OTM were trained on the same randomly selected subset of deaths and tested on the same randomly selected, non-overlapping subset of deaths. In order to derive uncertainty intervals for the mean performance, we resampled training and testing sets 35 times. For InterVA-4, we resampled testing sets 35 times, each time selecting 50 of the 555 records. These uncertainty intervals represent sampling probabilities and non-sampling errors in the performance of various methods.

To estimate the relevance of “local” learning (i.e., training classifiers on deaths from the same geographical region and hospital or non-hospital based setting as the test deaths), we trained NBC and OTM on both the PHMRC’s hospital-based VA dataset compiled from several countries [5, 20] and the MDS (using only non-hospital deaths), and tested on MDS (non-hospital) and PHMRC (hospital) deaths. In each case, the training and testing data sets contained 400 non-overlapping records. As with our other datasets, we resampled training and testing sets 35 times.

### Performance measures

At the individual level, we assessed performance using sensitivity, specificity, and partial chance-corrected concordance (PCCC). PCCC uses the proportion of true assigned CODs, while accounting for the number of possible COD groups [22]. At the population level, we used cause-specific mortality fraction (CSMF) accuracy to compare performance between the classifier- and physician-assigned COD distribution. CSMF accuracy is based on the absolute difference between estimated and actual CSMFs, and similarly accounts for the variation in the number of CODs across datasets [22]. We also examine sensitivity and specificity at the specific cause level for each classifier.

All analyses, data manipulation, and implementations of NBC and OTM were done in R [23]. The code is freely available in Additional file 2.

### Naive Bayes classifier

For a given VA record, NBC assigns a probability to each label (i.e., each COD), according to the (independent) conditional probabilities of that label given a specific feature (i.e., sign or symptom) and the unconditional probability of that same label. The label with the highest probability is then assigned to the VA record. In more detailed terms, we assign each record the cause of death *C*_j*_ with the highest probability (score), given a set of *n* recorded features (signs/symptoms) in the verbal autopsy, denoted by *F*_1_, …, *F*_*n*_:

1 $$ {C}_{j*}:= \arg \kern0.5em  \max {.}_j\kern0.5em \left\{ Pr\left({C}_j\left|{F}_1,\dots, {F}_n\right.\right)\right\} $$

where each of the features *F*_*i*_, are either 0, if the sign or symptom is not reported in the verbal autopsy, or 1, if it is.

Since we seek the cause with the highest probability, we can just focus on the following proportional relationship (for simplicity we use *Pr*(*F*_*i*_) to denote *Pr*(*F*_*i*_ = 1):

2 $$ Pr\left({C}_j\left|{F}_1,\dots, {F}_n\right.\right)\propto Pr\left({C}_j\right) Pr\left({F}_1,\dots, {F}_n\left|{C}_j\right.\right) $$

By applying the naive assumption, we obtain the following:

3 $$ Pr\left({C}_j\left|{F}_1,\dots, {F}_n\right.\right)\propto Pr\left({C}_j\right){\displaystyle {\prod}_{i=1}^n\left[{\rrbracket}_i Pr\left({F}_i\left|{C}_j\right.\right)+\left(1-{\rrbracket}_i\right)\left(1- Pr\left({F}_i\left|{C}_j\right.\right)\right)\right]} $$

where,

4 $$ Pr\left({C}_j\right)=\frac{\left|{c}_j\right|}{N} $$

5 $$ Pr\left({F}_i\left|{C}_j\right.\right)=\frac{Pr\left({F}_i\cap {C}_j\right)}{Pr\left({C}_j\right)}:=\frac{\left|{F}_i\cap {C}_j\right|/N}{\left|{C}_j\right|/N}=\frac{\left|{F}_i\cap {C}_j\right|}{\left|{C}_j\right|} $$

6 $$ {\rrbracket}_i=\left\{\begin{array}{c}\hfill 1\kern0.5em \mathrm{if}\kern0.5em \mathrm{feature}\kern0.5em i\kern0.5em \mathrm{is}\kern0.5em \mathrm{reported}\hfill \\0\kern0.5em \mathrm{otherwise}\hfill \end{array}\right. $$

and where, *n* is the total number of features (signs/symptoms), |*C*_*j*_| is the number of cases of death from cause *C*_*j*_ (in the training data set), |*F*_*i*_ ∩ *C*_*j*_| is the number of cases of death from cause *C*_*j*_ that exhibited feature *F*_*i*_ (also in the training data set), and *N* is the total number of observations in the training data set. The larger the size of the training data set, i.e. *N*, the closer the estimated *Pr*(*F*_*i*_|*C*_*j*_) is to the true probability of observing a feature (sign/symptom) given a particular COD.

### Comparison of InterVA-4, OTM, and NBC classifiers

InterVA-4 and OTM are two automated classification tools that use a symptom-cause scoring approach, similar to NBC. Table 2 compares the major features of these classifiers. OTM and NBC use a labelled training set to learn the distinguishing characteristics of each COD and apply them to unclassified data, while InterVA-4 uses static and exogenous probabilities provided by a panel of experts. These InterVA-4 probabilities cannot be considered as formal probabilities, from a mathematical standpoint, because they may lack mathematical consistency. On the other hand, in the case of the NBC, the probabilities of death from a given cause *C*_*j*_ (i.e., *Pr*(*C*_*j*_) and the conditional probabilities of displaying a sign or symptom *F*_*i*_, given a cause of death *C*_*j*_ (i.e., *Pr*(*F*_*i*_|*C*_*j*_)), are all estimated from a training dataset.

**Table 2:** Comparison of NBC to other VA classifiers

Feature	InterVA-4	OTM^a^	NBC
Learns from training set	No	Yes	Yes
Uses Bayes rule	Yes	No	Yes
Uses naive assumption	Yes	Yes	Yes
Accounts for absence of symptom	No	No	Yes

*NBC* naïve Bayes classifier, *VA* verbal autopsy, *OTM* open-source Tariff Method

^a^Our earlier publication demonstrates that the performance of our OTM to the original Tariff method is comparable [8]; the OTM performed almost exactly as the original Tariff method on the hospital-based dataset without the health care experience (HCE) variables (for the top cause), but less well than the same analysis with HCE variables. Note that results in the original Tariff publication without HCE were only available for the top assigned cause [7]. HCE variables are those that capture any information that the respondent may know about the decedent’s experiences with health care

All three classifiers rely on the counter-intuitive “naive” assumption that all signs or symptoms are independent, given a particular COD. For example, the probability of having a fever given one has pneumonia is assumed independent of the probability of having a cough given one has pneumonia. If a study collected information only for cough and fever, then NBC would use the training set of cases to estimate the probability of cough given pneumonia: Pr(cough|pneumonia) and the probability of fever given pneumonia: Pr(fever|pneumonia), as well as the probability of pneumonia: Pr(pneumonia) = (number of deaths due pneumonia)/(total number of deaths). NBC then calculates the overall prediction score that a particular COD is pneumonia, given both symptoms present, as: Pr(pneumonia)* Pr(cough|pneumonia) * Pr(fever|pneumonia), which will yield a high prediction score of pneumonia if the values of Pr(pneumonia), Pr(cough|pneumonia) and Pr(fever|pneumonia) are high. Despite its reliance on this counter-intuitive assumption of independent conditional probabilities, NBC has been shown to often outperform more sophisticated learning algorithms [10, 11].

InterVA-4 and OTM use the presence of a sign or symptom, while NBC additionally incorporates the absence of a sign or symptom in its scoring mechanism. Indeed, for both NBC and diagnosis by physicians, the *absence* of a sign or symptom may completely preclude a specific COD (e.g., absence of cough would likely exclude a diagnosis of respiratory tuberculosis death).

A final major difference between the algorithms lies in the way CODs are scored from most to least likely. InterVA-4 and NBC apply the Bayes theorem, while OTM assigns a weight (“tariff”) to each symptom pattern for a given COD. For example, cough would carry a much heavier weight for death due to pneumonia than for death due to diarrhea. The weights are then summed to produce a tariff score for each death and for each cause. OTM then assigns the cause with the highest tariff score as the COD for that individual. As the code for the Tariff algorithm is not freely available, we developed OTM which has performance comparable to the earlier published Tariff method [8].

Additional file 3 provides a further description of InterVA-4 and OTM.

## Results

Mean sensitivity and 95 % uncertainty intervals based on 35 train/test splits for individual-level performance of the three classifiers on the MDS, Agincourt, and Matlab datasets are presented in Table 3, and by detailed CODs in Additional files 4, 5, and 6. NBC and OTM achieved similar median sensitivity in all three datasets (median 0.51, range 0.48-0.58; and median 0.50, range 0.41-0.51, respectively). InterVA-4 achieved lower sensitivity (median 0.43, range 0.36-0.47). Similar trends were observed for partial chance-corrected concordance scores (Table 4). Specificity was consistently high for all three techniques across the datasets (median 0.96, range 0.96-0.97, data not shown). Specificity is designed to measure the performance of classifying elements into two groups, in this case two categories of COD, and hence, the reported specificities may be artificially high.

**Table 3:** Mean overall sensitivity (and 95 % uncertainty intervals) on three datasets for 35 train/test iterations

Study (training/testing sample size)^a^	NBC	OTM	InterVA-4^b^	Median, all three classifiers
MDS	0.57	0.50	0.43	0.50
(11,000/555)^c^	(0.57, 0.58)	(0.50,0.51)	(0.40,0.45)	
Agincourt	0.48	0.42	0.38	0.42
(2,300/2,300)	(0.48,0.48)	(0.41,0.42)	(0.36,0.41)	
Matlab	0.51	0.50	0.45	0.50
(1,000/1,000)	(0.50,0.51)	(0.50,0.51)	(0.43,0.47)	
Median, all three datasets	0.51	0.50	0.43	0.50

*NBC* naïve Bayes classifier, *OTM* open-source Tariff Method, *VA* verbal autopsy, *MDS* Million Death Study

^a^Training/testing sample size, with no training required for InterVA-4

^b^InterVA-4 was evaluated on a testing dataset of 50 randomly selected records out of 555 records, in each of the 35 iterations

^c^Sensitivity using 555/555 training/testing records from the MDS dataset were 0.55 (0.54, 0.55) and 0.49 (0.48, 0.50), respectively, for NBC and OTM

Specificity achieved by all automated classifiers across all datasets ranged from 0.96 to 0.97, and the largest uncertainty interval observed was (0.96,0.97)

**Table 4:** Partial chance-corrected concordance (and 95 % uncertainty intervals) on three datasets for 35 train/test iterations

Study (training/testing sample size)^a^	NBC	OTM	InterVA-4^b^	Median all three classifiers
MDS	0.54	0.47	0.39	0.47
(11,000/555)	(0.54, 0.55)	(0.46,0.47)	(0.36,0.41)	
Agincourt	0.43	0.38	0.34	0.38
(2,300/2,300)	(0.44,0.45)	(0.37,0.38)	(0.32,0.37)	
Matlab	0.47	0.47	0.41	0.47
(1,000/1,000)	(0.47,0.49)	(0.46,0.47)	(0.39,0.43)	
Median all three datasets	0.47	0.47	0.39	0.47

*NBC* naïve Bayes classifier, *OTM* open-source Tariff Method, *VA* verbal autopsy, *MDS* Million Death Study

^a^Training/testing sample size, with no training required for InterVA-4

^b^InterVA-4 was evaluated on a testing data set of 50 randomly selected records out of 555 records, in each of the 35 iterations

The NBC median accuracy was the most consistent, as defined by the fewest number of CODs scoring a sensitivity of zero (Table 5). The least consistent performance was for OTM. The NBC reported 2, 0, and 0 instances of zero sensitivity, and InterVA-4 reported 6, 1 and 1 such instances across the MDS, Agincourt and Matlab datasets, respectively. By contrast, OTM reported 10, 13 and 9 such instances across these three datasets, respectively. Additional files 4, 5 and 6 show these results by specific CODs.

**Table 5:** Number of instances with zero sensitivity for CODs

Study (number of CODs)	NBC	OTM	InterVA-4
MDS (15)	2	10	6
Agincourt (16)	0	13	1
Matlab (16)	0	9	1

*COD* cause of death, *NBC* naïve Bayes classifier, *OTM* open-source Tariff Method, *VA* verbal autopsy, *MDS* Million Death Study

Use of the larger MDS training set (11,000 records, compared to 555) yielded only marginal improvements in sensitivity and specificity for NBC and OTM (Table 3).

**Fig. 1:**
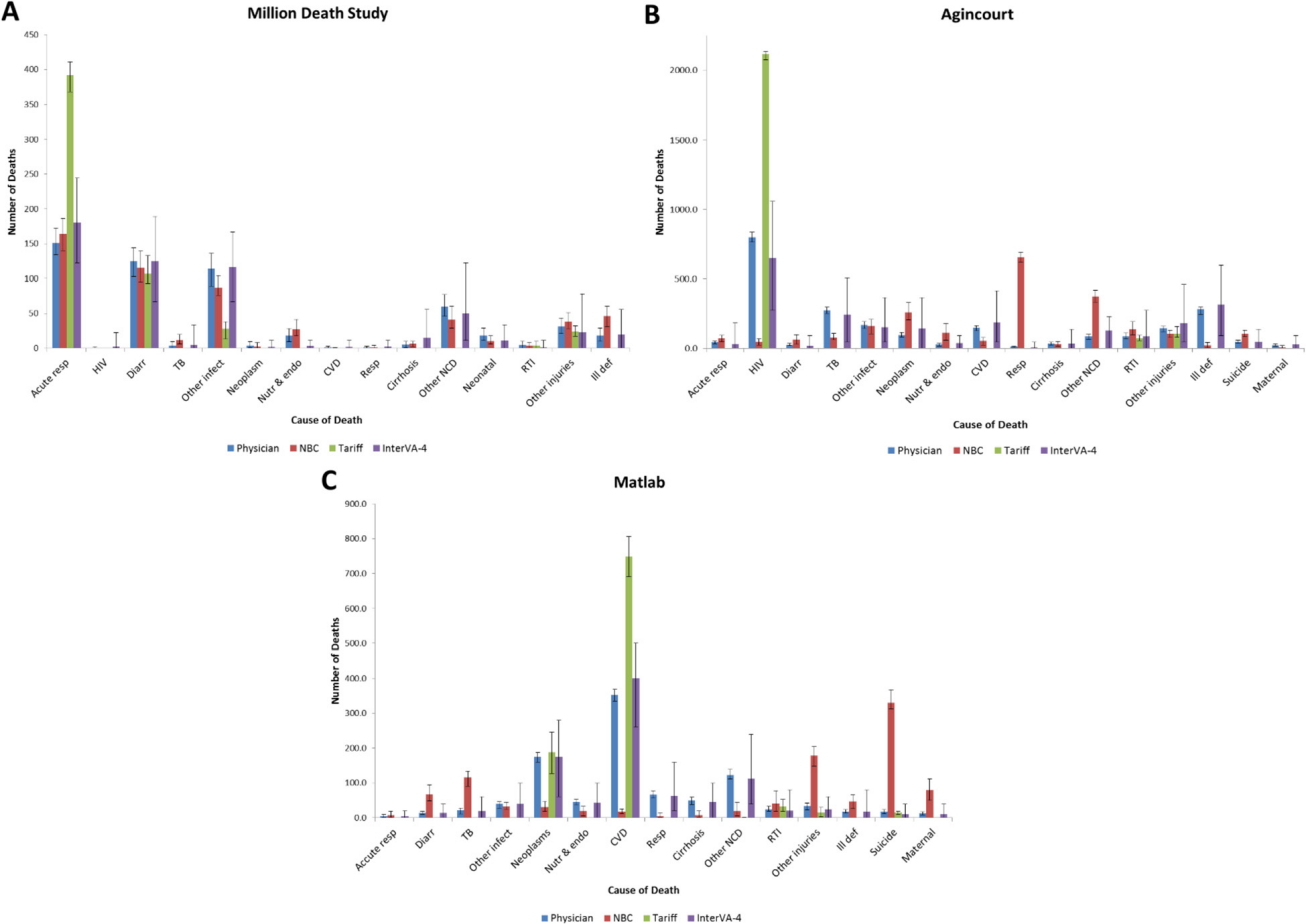
The mean, minimum, and maximum CSMFs as reported by the three classifiers across datasets for **a.** 15 causes using data from the Million Death Study, **b.** 16 causes using data from the Agincourt study, **c.** 15 causes using data from the Matlab study. The MDS results use 11,000 training cases and 555 test cases. *CSMF* cause-specific mortality fraction

Figure 1 shows the mean, minimum and maximum CSMFs as reported by the three classifiers across datasets. Since InterVA-4 was applied to a smaller subset of the original datasets, the frequency of assignments was scaled (multiplied by a constant) to make them comparable to the frequency of assignments of the other two classifiers (scaling constants were 555/50 for MDS, 2,300/50 for Agincourt, and 1,000/50 for Matlab). Compared to physician classification, NBC overestimated COD assignments for respiratory and tuberculosis deaths in the MDS data, and diarrhea, cancers, other non-communicable diseases and suicide in the Agincourt data; and diarrhea, tuberculosis, other injuries, ill-defined, suicide, and maternal in the Matlab data. Notable overestimation of CODs assigned by the OTM classifier were seen for acute respiratory deaths in children 1- to 59-months old in the MDS, HIV in the Agincourt data, and cardiovascular disease in the Matlab data (both among adults). InterVA-4 documented some HIV deaths among MDS child deaths while physicians did not, and similarly showed higher numbers of respiratory and cirrhosis deaths than physicians. InterVA-4 showed no notable overestimates in the Agincourt and Matlab datasets. Compared to physician classification, NBC underestimated cardiovascular diseases in all three datasets, while OTM underestimated ill-defined causes in all three datasets. Noticeable underestimates for InterVA-4 are only observed in the child dataset (MDS, namely for neoplasms and nutritional and endocrine CODs), and not in the adult datasets (Agincourt and Matlab).

Table 6 displays mean performance at the population level, using CSMF accuracy. NBC scored the highest median accuracy across the datasets (0.88, range 0.87-0.93), compared to InterVA-4 (0.66, range 0.62-0.73) and OTM (0.57, range 0.42-0.58). The CSMF accuracy of NBC on these data is comparable to previous results of the King-Lu method [8, 24], which is designed solely for population-level agreement [25].

**Table 6:** Mean CSMF accuracy (and 95 % uncertainty intervals) on three datasets for 35 train/test iterations

Study (training/testing sample size)^a^	NBC	OTM	InterVA-4^b^	Median all three classifiers
MDS	0.88	0.57	0.71	0.71
(11,000/555)	(0.87,0.88)	(0.56,0.57)	(0.69,0.73)	
Agincourt	0.87	0.42	0.66	0.66
(2,300/2,300)	(0.87,0.88)	(0.42,0.43)	(0.63,0.68)	
Matlab	0.92	0.57	0.65	0.65
(1,000/1,000)	(0.92,0.93)	(0.56,0.58)	(0.62,0.67)	
Median all three datasets	0.88	0.57	0.66	0.66

*CSMF* cause-specific mortality fraction, *NBC* naïve Bayes classifier, *OTM* open-source Tariff Method, *VA* verbal autopsy, *MDS* Million Death Study

^a^Training/testing sample size, with no training required for InterVA-4

^b^InterVA-4 was evaluated on a testing data set of 50 randomly selected records out of 555 records, in each of the 35 iterations

NBC and OTM each showed better accuracy on the test data when using training data from the same context (i.e., hospital deaths versus non-hospital deaths) as the test data (Table 7). When testing on the MDS data, training on the PHMRC data yielded sensitivity of 0.50 with NBC and 0.41 with OTM; conversely, training on the MDS yielded a sensitivity of 0.61 with NBC and 0.54 with OTM. Similarly, when testing on PHMRC data, training with MDS data had worse performance (NBC = 0.37 sensitivity; OTM = 0.32 sensitivity), than when training with PHMRC data (NBC sensitivity = 0.46; OTM sensitivity =0.40).

**Table 7:** Mean sensitivity (and 95 % uncertainty intervals) for various non-hospital deaths (MDS) and hospital deaths (PHMRC) train/test combinations for 35 train/test iterations

Train-test combination	NBC	OTM
MDS-MDS	0.61	0.54
	(0.60,0.62)	(0.52,0.55)
PHMRC-MDS	0.50	0.41
	(0.49,0.51)	(0.40,0.42)
PHMRC-PHMRC	0.46	0.40
	(0.45,0.47)	(0.38,0.41)
MDS-PHMRC	0.37	0.32
	(0.36,0.39)	(0.31,0.34)

Note: We selected 400 records for training and testing, respectively, in each of the 35 iterations. MDS cases used in this table are non-hospital based deaths, while PHMRC are hospital-based deaths. *MDS* Million Death Study, *PHMRC* Population Health Metrics Research Consortium

## Discussion

This is, to our knowledge, the first study to introduce NBC as a technique to classify VA deaths, and to compare its performance to the OTM and InterVA-4 classifiers. Our paper’s aim was to assess if automated methods can at least reproduce physician coding, rather than validating the assignment of true CODs. This study used datasets from three distinct epidemiological settings, covering 21,000 deaths, to test these classifiers. Using physician classification as the reference standard, we found that NBC substantially outperforms OTM and InterVA-4 at the population level, although the performance for all three methods at the individual level is modest at best. We also found that NBC yields the most consistent scoring, with few individual CODs showing a sensitivity of zero, while OTM was the least consistent.

One possible explanation for our results is that NBC and OTM have an advantage over InterVA-4 due to their ability to train on a subset of the data. Unlike OTM, NBC also uses the underlying proportion of CODs in the training set in its decision-making. This accounting may explain NBC’s particularly good performance at the population level. Indeed, NBC performance at the population level rivalled that of the King-Lu method [8, 24] and has the advantage of being able to assign individual CODs, which King-Lu does not. However, if CODs have peculiar sign or symptom patterns, but low prevalence in the training data, accounting for underlying COD prevalence may result in erroneous classification with both NBC and King-Lu. InterVA-4 has recently claimed population-level agreement of over 80 % on health and demographic data, after several adjustments [26]. However, such performance has not been seen on the datasets here or in our earlier analyses [8], nor when run on the PHMRC hospital-based dataset [27]. NBC specifically accounts for the absence of a sign or symptom, unlike OTM and InterVA-4, which may improve its diagnostic accuracy. Finally, while our OTM is not identical to the published Tariff method, its performance is very similar, as we have previously demonstrated [8]. We do not believe that these minor differences reduced the robustness of our findings.

A recently described method, InSilicoVA, claims to attain much higher sensitivity at the individual level when trained and tested on the hospital-based PHMRC dataset [27]. Nevertheless, performance on deaths out of hospital is not likely to be comparable. Overall, automated classifiers' sensitivity in matching physician coding at the individual level generally remains low. In all of the training and testing scenarios in this study, the automated classifiers are validated such that the testing set COD distribution is similar to the training set, and not other COD distributions. As such, performance might be overestimated.

Automated classification does not yet seem to present a viable substitute for physician classification, especially at the individual level [8, 9]. Our comparison standard of physician-assigned CODs has inherent errors [3, 5, 6, 8], but remains the most widely accepted clinical standard worldwide. Barring major improvement in automated classifiers matching individual physician codes, current computer methods will not likely meet the needs of plans to achieve universal registration of all deaths with medical certification [28, 29]. Indeed, the social acceptability of households accepting a machine certified COD (or probabilities) as official death certification is likely to be low.

It has been claimed that the Tariff method performs better than physicians on a hospital-based clinical dataset and it is implied that this hospital-based clinical dataset enables use of the Tariff classifier across settings [5]. Moreover, an online tool for Android phones to capture information needed for the Tariff has been promoted [7] and might be used in the on-going Bloomberg Data for Health Initiative [30]. We believe that such use is premature for three reasons. First, hospital datasets may not be representative of the recorded symptomatology and pathogen distribution of rural or medically-unattended deaths [4, 20, 31]. Second, the current tool claims good performance without any local training requirement. However, we reach the opposite conclusion. Indeed, even within the PHMRC hospital-based dataset, InSilicoVA trained on one country and tested on another performed notably worse [27]. Finally, the consistency of the OTM method was the lowest of our three classifiers, suggesting that cross-country and cross-place of death (hospital versus non-hospital) comparisons might be problematic [5, 20, 32].

Physician-based classification has obvious limitations [3, 5]. To address some of these, the MDS has adopted a web-based platform with dual, anonymous coding by physicians with strict guidelines, differential diagnosis and other quality control checks to improve reproducibility [4]. The web-based portal also enables large numbers of physicians (currently 400) to code at low cost: about USD$0.75 per record. Further enhancements might include merging physician and computer coding, as well as the development of a classifier that does not rely on the naive assumption. Further evaluation of these automated classifiers using several different testing methodologies and VA datasets would also be beneficial to the field.

VA methods may also benefit from dimensionality reduction in the features, reflected in a more focused questionnaire, as well as testing the possibility of employing a combination of algorithms and various statistical techniques [33]. We saw some evidence that the OTM worked better than the other classifiers for VA deaths where there were large differences between the tariff scores of the leading and second-leading predicted COD (for example, in areas dominated by HIV/AIDS deaths; data not shown). We, along with the International Institute of Population Sciences in India, are conducting several randomized comparisons of a narrative-based tool used in the MDS and a longer, symptom-list driven questionnaire akin to the WHO VA tool (F Ram, personal communication). This randomized evidence should provide important insights on choice of computer versus physician coding.

## Conclusions

This study has demonstrated that at the individual level, sensitivity remains low for automated VA classifiers, including for NBC. Hence, significant improvements are required before these techniques can be considered as adequate substitutes for physician classification.

### Ethical approval

Ethics approval for the Million Death Study was obtained from the Post Graduate Institute of Medical Research, St. John’s Research Institute and St. Michael’s Hospital, Toronto, Ontario, Canada.

Ethical clearance for health and demographic surveillance in Agincourt was granted by the University of the Witwatersrand’s Committee for Research on Human Subjects (Medical).

All individuals gave written informed consent to participate in the Matlab study. An icddrb institutional review committee and the icddrb Ethical Review Committee approved the baseline study.

The verbal autopsy procedures at each PHMRC study site followed the relevant hospital’s ethics board.

## Additional files

Additional file 1:
**Mapping of WHO cause of death categories. **(DOC 50 kb) Additional file 2:
**R code used to produce the results in this study. **(ZIP 101 kb) Additional file 3:
**Description of InterVA-4 and Open-source Tariff Method. **(DOC 25 kb) Additional file 4:
**Sensitivity and specificity of assignment by cause of death, on MDS data (ages 1–59 months). **(DOC 69 kb) Additional file 5:
**Sensitivity and specificity of assignment by cause of death, on Agincourt data (ages 15–64 years). **(DOC 71 kb) Additional file 6:
**Sensitivity and specificity of assignment by cause of death, on Matlab data (ages 20–64 years).** (DOC 69 kb)

## Abbreviations

COD: Cause of death
CSMF: Cause-specific mortality fraction
MDS: Million Death Study
NBC: Naive Bayes classifier
OTM: Open-source Tariff Method
PHMRC: Population Health Metrics Research Consortium
VA: Verbal autopsy
