# Supplementary material for: Naive Bayes classifiers for verbal autopsies: comparison to physician-based classification for 21,000 child and adult deaths
Source: BMC Med. 2015 Nov 25;13:286. doi: 10.1186/s12916-015-0521-2 (PMC4660822; doi:10.1186/s12916-015-0521-2)
Supplement: Additional file 1: — Mapping of WHO cause of death categories. (DOC 50 kb) [file 12916_2015_521_MOESM1_ESM.doc]

**Additional file 1: Mapping of WHO cause of death categories**

| **Abbreviation** | **Label** |  |  | **WHO categories** | | | |  |  |
| --- | --- | --- | --- | --- | --- | --- | --- | --- | --- |
|  |  |  |  |  |  |  |  |  |  |
| Acute resp | Acute respiratory | 1.02 |  |  |  |  |  |  |  |
| HIV | HIV/AIDS | 1.03 |  |  |  |  |  |  |  |
| Diarr | Diarrhoeal | 1.04 |  |  |  |  |  |  |  |
| TB | Pulmonary TB | 1.09 |  |  |  |  |  |  |  |
| Other infect | Other and unspecified infections | 1.05 | 1.06 | 1.07 | 1.08 | 1.01 | 1.1 | 1.11 | 1.99 |
| Neoplasm | Neoplasms/cancers | 2.01 | 2.02 | 2.03 | 2.04 | 2.05 | 2.06 | 2.99 |  |
| Nutr & endo | Nutritional and endocrine | 3.01 | 3.02 | 3.03 |  |  |  |  |  |
| CVD | Cardio-vascular disease | 4.01 | 4.02 | 4.99 |  |  |  |  |  |
| Resp | Respiratory disorders | 5.01 | 5.02 |  |  |  |  |  |  |
| Cirrhosis | Liver cirrhosis | 6.02 |  |  |  |  |  |  |  |
| Other NCD | Other non-communicable disease | 7.01 | 8.01 | 98 | 10.06 | 4.03 | 6.01 |  |  |
| Neonatal | Neonatal conditions | 10.01 | 10.02 | 10.03 | 10.04 | 10.05 | 10.99 |  |  |
| RTI | Road and transport injuries | 12.01 | 12.02 |  |  |  |  |  |  |
| Other injuries | Other injuries | 12.03 | 12.04 | 12.05 | 12.06 | 12.07 | 12.09 | 12.1 | 12.99 |
| Ill def | Ill-defined | 99 |  |  |  |  |  |  |  |
| Suicide | Suicide | 12.08 |  |  |  |  |  |  |  |
| Maternal | Maternal | 9.01 | 9.02 | 9.03 | 9.04 | 9.05 | 9.06 | 9.07 | 9.08 9.99 |
|  |  |  |  |  |  |  |  |  |  |
