# Supplementary material for: Naive Bayes classifiers for verbal autopsies: comparison to physician-based classification for 21,000 child and adult deaths
Source: BMC Med. 2015 Nov 25;13:286. doi: 10.1186/s12916-015-0521-2 (PMC4660822; doi:10.1186/s12916-015-0521-2)

README

Overview: The R programs provided in this folder evaluate machine learning classifiers for verbal autopsies. The classifiers include

Naive-Bayes Classification (NBC) and Open Source Tariff (OST).

R programs included:

1) ranID.r

2) mainMaster.r

3) mainNBC.r

4) mainTariff.r

**Instructions**

**Step 1:** Prepare verbal autopsy (VA) datasets

VA datasets must be a .csv and have the following format:

**Column 1** **–** unique identifier (ID)

**Column 2** **–** Gold-standard Cause of Death (COD) number (i.e. 1 = pneumonia, 2 = HIV, etc.)

**Column 3 to Mth column** **–** symptoms (1 = occurrence, 0 = no occurrence)

**Row 1** – Variable labels

**Row 2 to Nth row** **–** Observations


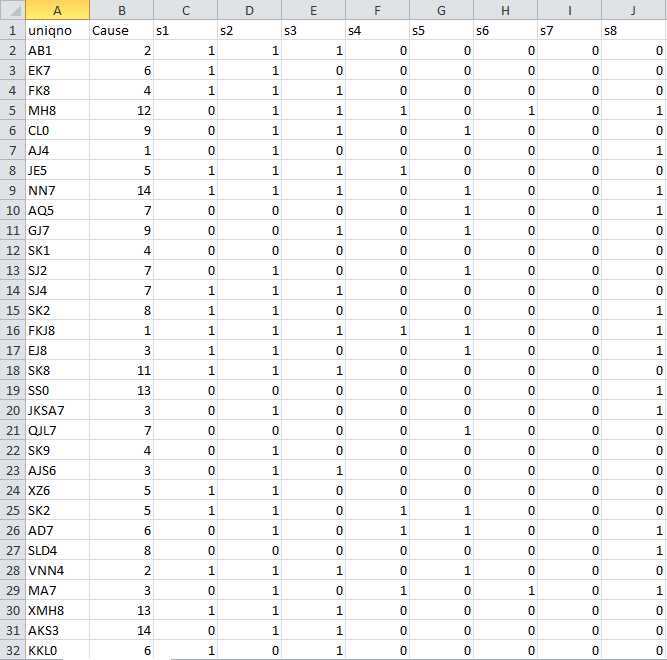


**Step 2:** Run ranID.r

ranID.r creates .csv files consisting of randomly selected observations for the training and testing sets. These files are eventually called in mainMaster.r. Run ranID.r by completing the required inputs specified in the code.

The output files should be stored in a folder that will be called by mainMaster.r.

**Step 3:** Run mainMaster.r

Complete the required inputs specified in mainMaster.r and run the code. Ensure that mainNBC and mainTariff are in the same folder as the working directory. The NBC and OST results (resultsNBC and resultsTariff) will be converted to a .csv and exported to the specified output directory. The results have the following format:

**Column 1 –** Total false negatives

**Column 2 –** Total false positives

**Column 3 –** Total true negatives

**Column 4 –** Total true positives

**Column 5 –** Sensitivity

**Column 6 –** Specificity

**Column 7 –** PCCC

**Column 8 –** CSMF Accuracy

**Row 1 to Nth row –** Iteration number


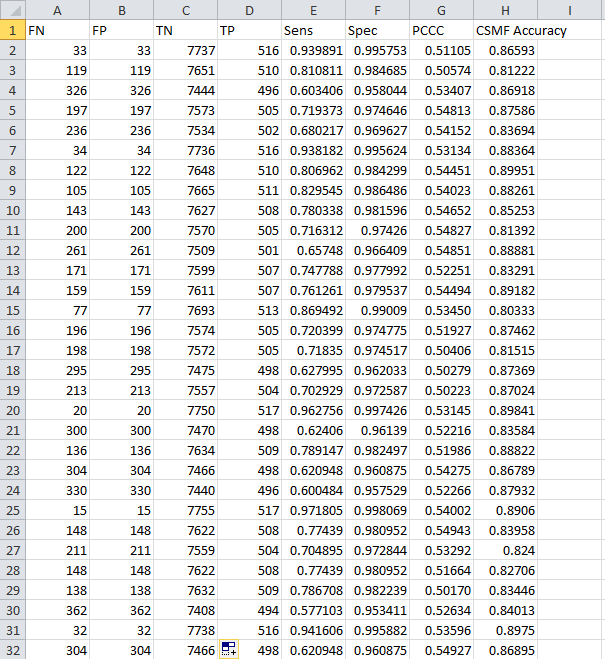

Supplement: Additional file 2: — R code used to produce the results in this study. (ZIP 101 kb) [file 12916_2015_521_MOESM2_ESM.zip › AdditionalFile2_vFINAL2/README.docx]
