# Supplementary material for: Naive Bayes classifiers for verbal autopsies: comparison to physician-based classification for 21,000 child and adult deaths
Source: BMC Med. 2015 Nov 25;13:286. doi: 10.1186/s12916-015-0521-2 (PMC4660822; doi:10.1186/s12916-015-0521-2)
Supplement: Additional file 3: — Description of InterVA-4 and Open-source Tariff Method. (DOC 25 kb) [file 12916_2015_521_MOESM3_ESM.doc]

**Additional file 3: Description of InterVA-4 and Open-source Tariff Method (OTM)**

***InterVA-4***

InterVA-4 is a pseudo-probabilistic model that assigns a cause of death to a verbal autopsy record, on the basis of recorded signs and symptoms. It was developed and presented by [16], which provide full description of this technique.

InterVA-4 uses a scoring system roughly based on Bayes’ rule, with propensities in lieu of formal probabilities. The propensity that a person died of a cause given a set of symptoms is computed using the likelihood of a symptom given a particular cause of death. The propensity of symptom given a cause is obtained from an exogenous, static group of physicians. The algorithm used to compute scores for InterVA-4 contains inconsistencies based on the symptom set solicited from physicians and only uses the presence of a symptom, without explicitly accounting for the effect of its absence.

Unlike Tariff and the naive Bayes classifier, InterVA-4 requires no training or any sort of input-parameter estimation by the end-user [16]. All the necessary probabilities are embedded in the software, which makes it extremely valuable as a starting point for analyses in situations where already classified verbal autopsy data is unavailable. Additionally, earlier versions of InterVA-4 have been shown to be robust to variations (often due to estimation error) of the a priori probabilities, for population level cause of death estimates (Fottrell, E., et al, 2011 http://journals.plos.org/plosone/article?id=10.1371/journal.pone.0027200).

For our purpose, it is important to note the following. Each sign or symptom and cause of death pair has a corresponding conditional “probability." Each cause of death also has an unconditional “probability". These “probabilities" are assigned by knowledgeable area experts using a qualitative scale and then embedded in the software. The final assigned cause of death is the cause that yields the highest “probability" (propensity), given the deceased's signs and symptoms, as recorded in the verbal autopsy [16].

Finally, it should be remembered that because these probabilities are not estimated directly from a data set, but instead are assigned qualitatively, they are not formally probabilities. The reader should be aware that we use the term “probability" loosely, in the case of InterVA-4 (this detail explains the use of quotations around probability and the use of the term “propensity" as a synonym).

***Open-source Tariff Method (OTM)***

Tariff is a learning-based tool for classifying verbal autopsies. It was developed by the University of Washington's IHME and presented in [7]. It does not assign a probability to each possible cause of death. Instead, it classifies death records according to the similarity between their recorded signs and symptoms and those of a typical case from each of the possible causes.

This typical pattern of signs and symptoms is obtained from the training data set, which is why Tariff is referred to as a learning algorithm. Similarity to each cause pattern is measured through a (0-1) weighted sum of the tariffs for each symptom-cause pair recorded in the autopsy, the “Tariff score". These tariffs are analogous to standardized residuals.

The Tariff code is not freely available to run on data not captured through the open-source SMARTVA application (http://www.healthdata.org/verbal-autopsy/tools), for example, MDS, Agincourt and Matlab datasets. In this study we use our version of Tariff, the OTM. In our previous work we have demonstrated that results achieved by OTM are comparable to that of Tariff [8].
