# Supplementary material for: Naive Bayes classifiers for verbal autopsies: comparison to physician-based classification for 21,000 child and adult deaths
Source: BMC Med. 2015 Nov 25;13:286. doi: 10.1186/s12916-015-0521-2 (PMC4660822; doi:10.1186/s12916-015-0521-2)
Supplement: Additional file 4: — Sensitivity and specificity of assignment by cause of death, on MDS data (ages 1–59 months). (DOC 69 kb) [file 12916_2015_521_MOESM4_ESM.doc]

**Additional file 4: Sensitivity and specificity of assignment by cause of death on MDS data (ages 1-59 months)**

|  | **InterVA-4** | | **OTM** | | **Naïve Bayes** | |
| --- | --- | --- | --- | --- | --- | --- |
|  | NA/50 | | 11,000/555 | | 11,000/555 | |
| **COD** | **Sens.** | **Spec.** | **Sens.** | **Spec.** | **Sens.** | **Spec.** |
| Acute resp | 46.90% | 86.60% | 98.40% | 39.80% | 74.80% | 87.50% |
|  | (43.2% - 50.7%) | (84.8% - 88.3%) | (98.0% - 98.8%) | 39.0% - 40.5%) | (73.9% - 75.8%) | (86.9% - 88.1%) |
| HIV | 0.00% | 93.10% | 0.00% | 100.00% | 0.00% | 100.00% |
|  | (0.0% - 0.0%) | (91.9% - 94.2%) | (0.0% - 0.0%) | (100.0% - 100.0%) | (0.0% - 0.0%) | (100.0% - 100.0%) |
| Diarr | 51.10% | 90.70% | 64.50% | 93.70% | 70.20% | 93.40% |
|  | (45.9% - 56.2%) | (89.4% - 91.9%) | (63.1% - 66.0%) | (93.3% - 94.1%) | (68.9% - 71.5%) | (93.0% - 93.8%) |
| TB | 41.70% | 98.80% | 0.00% | 100.00% | 24.30% | 98.10% |
|  | (26.2% - 57.1%) | (98.4% - 99.2%) | (0.0% - 0.0%) | (100.0% - 100.0%) | (16.3% - 32.4%) | (97.9% - 98.3%) |
| Other infect | 51.40% | 67.20% | 21.70% | 99.30% | 47.20% | 92.50% |
|  | (47.6% - 55.1%) | (64.6% - 69.7%) | (20.4% - 23.1%) | (99.2% - 99.5%) | (46.0% - 48.4%) | (92.1% - 92.9%) |
| Neoplasm | 0.00% | 100.00% | 0.00% | 100.00% | 4.20% | 99.50% |
|  | (0.0% - 0.0%) | (100.0% - 100.0%) | (0.0% - 0.0%) | (100.0% - 100.0%) | (0.5% - 8.0%) | (99.4% - 99.6%) |
| Nutr & endo | 55.60% | 95.50% | 0.00% | 100.00% | 41.80% | 96.30% |
|  | (38.1% - 73.0%) | (94.7% - 96.4%) | (0.0% - 0.0%) | (100.0% - 100.0%) | (37.9% - 45.8%) | (96.0% - 96.6%) |
| CVD | 40.00% | 99.80% | 0.00% | 100.00% | 0.00% | 99.90% |
|  | (21.9% - 58.1%) | (99.6% - 100.0%) | (0.0% - 0.0%) | (100.0% - 100.0%) | (0.0% - 0.0%) | (99.9% - 100.0%) |
| Resp | 0.00% | 99.90% | 0.00% | 100.00% | 15.40% | 99.80% |
|  | (0.0% - 0.0%) | (99.7% - 100.0%) | (0.0% - 0.0%) | (100.0% - 100.0%) | (4.1% - 26.6%) | (99.8% - 99.9%) |
| Cirrhosis | 0.00% | 100.00% | 0.00% | 100.00% | 18.20% | 99.00% |
|  | (0.0% - 0.0%) | (100.0% - 100.0%) | (0.0% - 0.0%) | (100.0% - 100.0%) | (10.3% - 26.2%) | (98.8% - 99.1%) |
| Other NCD | 13.30% | 97.80% | 0.00% | 100.00% | 22.50% | 94.50% |
|  | (7.8% - 18.8%) | (97.2% - 98.4%) | (0.0% - 0.0%) | (100.0% - 100.0%) | (20.6% - 24.4%) | (94.1% - 94.9%) |
| Neonatal | 0.00% | 100.00% | 0.00% | 100.00% | 15.10% | 98.60% |
|  | (0.0% - 0.0%) | (100.0% - 100.0%) | (0.0% - 0.0%) | (100.0% - 100.0%) | (11.9% - 18.3%) | (98.3% - 98.8%) |
| RTI | 100.00% | 100.00% | 80.00% | 99.90% | 75.50% | 99.90% |
|  | (100.0% - 100.0%) | (100.0% - 100.0%) | (72.2% - 87.7%) | (99.9% - 100.0%) | (67.5% - 83.5%) | (99.9% - 100.0%) |
| Other injuries | 80.60% | 99.80% | 69.90% | 99.50% | 79.30% | 97.40% |
|  | (70.9% - 90.3%) | (99.6% - 100.0%) | (67.5% - 72.3%) | (99.4% - 99.6%) | (77.2% - 81.4%) | (97.2% - 97.6%) |
| Ill def | 0.00% | 99.80% | 0.00% | 100.00% | 47.60% | 93.00% |
|  | (0.0% - 0.0%) | (99.6% - 100.0%) | (0.0% - 0.0%) | (100.0% - 100.0%) | (43.9% - 51.2%) | (92.6% - 99.3%) |
| **OVERALL** | **42.60%** | **95.90%** | **50.20%** | **96.40%** | **57.00%** | **96.90%** |
|  | **(40.0% - 45.2%)** | **(95.7% 96.1%)** | **49.7% - 50.7%)** | **(96.4% - 96.5%)** | **(56.6% - 57.5%)** | **(96.9% - 97.0%)** |

Acute resp=acute respiratory; TB=pulmonary TB; Other infect.=other and unspecified infections; Nutr. & endo.=nutrition and endocrine; Cirrhosis=live cirrhosis; RTI=road and transport injuries.
