# Supplementary material for: Naive Bayes classifiers for verbal autopsies: comparison to physician-based classification for 21,000 child and adult deaths
Source: BMC Med. 2015 Nov 25;13:286. doi: 10.1186/s12916-015-0521-2 (PMC4660822; doi:10.1186/s12916-015-0521-2)
Supplement: Additional file 5: — Sensitivity and specificity of assignment by cause of death, on Agincourt data (ages 15–64 years). (DOC 71 kb) [file 12916_2015_521_MOESM5_ESM.doc]

**Additional file 5: Sensitivity and specificity of assignment by cause of death, on Agincourt data (ages 15-64 years)**

|  | **InterVA-4** | | **OTM** | | **Naïve Bayes** | |
| --- | --- | --- | --- | --- | --- | --- |
|  | **NA/50** | | **2,300/2,300** | | **2,300/2,300** | |
| **COD** | **Sens.** | **Spec.** | **Sens.** | **Spec.** | **Sens.** | **Spec.** |
| Acute resp | 53.90% | 92.50% | 0.00% | 100.00% | 31.60% | 97.30% |
|  | (37.8% - 70.1%) | (91.1% - 93.9%) | (0.0% - 0.0%) | (100.0% - 100.0%) | (29.6% - 33.6%) | (97.2% - 97.5%) |
| HIV | 45.90% | 80.50% | 100.00% | 12.10% | 60.10% | 88.40% |
|  | (41.2% - 50.6%) | (78.3% - 82.8%) | (100.0% - 100.0%) | (11.7% - 12.6%) | (59.6% - 60.7%) | (88.1% - 88.7%) |
| Diarr | 8.30% | 99.40% | 0.00% | 100.00% | 22.80% | 98.80% |
|  | (0.0% - 17.9%) | (99.0% - 99.7%) | (0.0% - 0.0%) | (100.0% - 100.0%) | (20.1% - 25.4%) | (98.7% - 98.9%) |
| TB | 75.70% | 84.00% | 0.00% | 100.00% | 58.20% | 89.40% |
|  | (69.2% - 82.1%) | (82.3% - 85.7%) | (0.0% - 0.0%) | (100.0% - 100.0%) | (57.1% - 59.4%) | (89.1% - 89.6%) |
| Other infect | 13.70% | 98.40% | 0.00% | 100.00% | 29.40% | 95.80% |
|  | (5.2% - 22.1%) | (97.8% - 98.9%) | (0.0% - 0.0%) | (100.0% - 100.0%) | (28.0% - 30.8%) | (95.6% - 96.1%) |
| Neoplasm | 33.90% | 92.00% | 0.00% | 100.00% | 33.30% | 96.70% |
|  | (23.4% - 44.5%) | (90.9% - 93.2%) | (0.0% - 0.0%) | (100.0% - 100.0%) | (31.8% - 34.9%) | (96.5% - 96.8%) |
| Nutr & endo | 9.50% | 98.00% | 0.00% | 100.00% | 30.70% | 99.40% |
|  | (1.0% - 18.0%) | (97.3% - 98.7%) | (0.0% - 0.0%) | (100.0% - 100.0%) | (27.2% - 34.1%) | (99.3% - 99.5%) |
| CVD | 29.60% | 95.40% | 0.00% | 100.00% | 29.00% | 97.00% |
|  | (22.7% - 36.4%) | (94.4% - 96.5%) | (0.0% - 0.0%) | (100.0% - 100.0%) | (28.0% - 30.0%) | (96.9% - 97.1%) |
| Resp | 66.70% | 97.10% | 0.00% | 100.00% | 17.90% | 99.60% |
|  | (49.6% - 83.8%) | (96.4% - 97.8%) | (0.0% - 0.0%) | (100.0% - 100.0%) | (13.9% - 21.8%) | (99.6% - 99.7%) |
| Cirrhosis | 6.30% | 99.60% | 0.00% | 100.00% | 29.20% | 98.30% |
|  | (0.6% - 11.9%) | (99.3% - 99.9%) | (0.0% - 0.0%) | (100.0% - 100.0%) | (26.9% - 31.5%) | (98.2% - 98.5%) |
| Other NCD | 7.00% | 97.60% | 0.00% | 100.00% | 17.10% | 97.80% |
|  | (2.4% - 11.7%) | (96.9% - 98.4%) | (0.0% - 0.0%) | (100.0% - 100.0%) | (15.6% - 18.7%) | (97.6% - 98.0%) |
| RTI | 87.50% | 99.60% | 83.30% | 99.80% | 78.50% | 99.50% |
|  | (79.6% - 95.4%) | (99.4% - 99.9%) | (82.1% - 84.4%) | (99.8% - 99.8%) | (77.4% - 79.6%) | (99.3% - 99.6%) |
| Other injuries | 80.90% | 96.70% | 61.00% | 99.20% | 64.10% | 96.80% |
|  | 73.9% - 87.9%) | (95.9% - 97.5%) | (58.7% - 63.3%) | (99.0% - 99.4%) | (62.0% - 66.3%) | (96.5% - 97.1%) |
| Ill def | 0.00% | 100.00% | 0.00% | 100.00% | 28.10% | 90.90% |
|  | (0.0% - 0.0%) | (100.0% - 100.0%) | (0.0% - 0.0%) | (100.0% - 100.0%) | (27.2% - 29.0%) | (90.6% - 96.6%) |
| Suicide | 11.80% | 98.80% | 0.00% | 100.00% | 60.00% | 96.30% |
|  | (2.0% - 21.6%) | (98.2% - 99.3%) | (0.0% - 0.0%) | (100.0% - 100.0%) | (56.0% - 64.0%) | (96.0% - 96.6%) |
| Maternal | 25.00% | 99.80% | 0.00% | 100.00% | 46.20% | 98.00% |
|  | (10.2% - 39.8%) | (99.6% - 100.0%) | (0.0% - 0.0%) | (100.0% - 100.0%) | (42.3% - 50.2%) | (97.9% - 98.2%) |
| **OVERALL** | **38.30%** | **95.90%** | **41.70%** | **96.10%** | **47.80%** | **96.50%** |
|  | **(35.9% - 40.8%)** | **(95.7% - 96.1%)** | **(41.4% - 42.0%)** | **(96.1% - 96.1%)** | **(47.5% - 48.0%)** | **(96.5% - 96.5%)** |

Acute resp=acute respiratory; TB=pulmonary TB; Other infect.=other and unspecified infections; Nutr. & endo.=nutrition and endocrine; Cirrhosis=live cirrhosis; RTI=road and transport injuries.
