# Supplementary material for: Naive Bayes classifiers for verbal autopsies: comparison to physician-based classification for 21,000 child and adult deaths
Source: BMC Med. 2015 Nov 25;13:286. doi: 10.1186/s12916-015-0521-2 (PMC4660822; doi:10.1186/s12916-015-0521-2)
Supplement: Additional file 6: — Sensitivity and specificity of assignment by cause of death, on Matlab data (ages 20–64 years). (DOC 69 kb) [file 12916_2015_521_MOESM6_ESM.doc]

**Additional file 6: Sensitivity and specificity of assignment by cause of death on Matlab data (ages 20-64 years)**

|  | **InterVA-4** | | **OTM** | | **Naïve Bayes** | |
| --- | --- | --- | --- | --- | --- | --- |
|  | **NA/50** | | **1,000/1,000** | | **1,000/1,000** | |
| **COD** | **Sens.** | **Spec.** | **Sens.** | **Spec.** | **Sens.** | **Spec.** |
| Accute resp | 33.30% | 94.80% | 0.00% | 100.00% | 3.30% | 99.30% |
|  | (16.8% - 49.9%) | (93.7% - 95.9%) | (0.0% - 0.0%) | (100.0% - 100.0%) | (0.5% - 6.2%) | (99.2% - 99.4%) |
| Diarr | 21.10% | 99.70% | 0.00% | 100.00% | 13.00% | 99.40% |
|  | (8.3% - 33.8%) | (99.3% - 100.0%) | (0.0% - 0.0%) | (100.0% - 100.0%) | (10.1% - 15.9%) | (99.3% - 99.6%) |
| TB | 86.40% | 86.00% | 0.00% | 100.00% | 18.10% | 98.40% |
|  | (77.1% - 95.6%) | (84.5% - 87.5%) | (0.0% - 0.0%) | (100.0% - 100.0%) | (15.2% - 20.9%) | (98.1% - 98.6%) |
| Other infect | 13.20% | 97.20% | 0.00% | 100.00% | 10.40% | 96.00% |
|  | (5.8% - 20.6%) | (96.4% - 98.1%) | (0.0% - 0.0%) | (100.0% - 100.0%) | (8.9% - 11.8%) | (95.7% - 96.4%) |
| Neoplasms | 79.50% | 82.40% | 62.30% | 90.40% | 60.60% | 91.20% |
|  | (74.0% - 85.1%) | (80.6% - 84.2%) | (59.7% - 64.9%) | (89.6% - 91.3%) | (59.3% - 62.0%) | (90.7% - 91.6%) |
| Nutr & endo | 26.00% | 96.80% | 0.00% | 100.00% | 29.90% | 96.60% |
|  | (15.0% - 37.0%) | (96.1% - 97.4%) | (0.0% - 0.0%) | (100.0% - 100.0%) | (28.1% - 31.7%) | (96.3% - 96.8%) |
| CVD | 48.60% | 93.50% | 98.40% | 37.80% | 69.00% | 86.60% |
|  | (44.6% - 52.7%) | (92.1% - 94.8%) | (98.2% - 98.6%) | (36.3% - 39.3%) | (68.2% - 69.7%) | (86.2% - 87.1%) |
| Resp | 22.10% | 93.70% | 0.00% | 100.00% | 60.90% | 95.80% |
|  | (12.6% - 31.6%) | (92.3% - 95.1%) | (0.0% - 0.0%) | (100.0% - 100.0%) | (58.5% - 63.3%) | (95.4% - 96.1%) |
| Cirrhosis | 7.20% | 99.50% | 0.00% | 100.00% | 43.90% | 95.20% |
|  | (0.4% - 14.0%) | (99.1% - 99.8%) | (0.0% - 0.0%) | (100.0% - 100.0%) | (41.5% - 46.4%) | (94.8% - 95.6%) |
| Other NCD | 19.30% | 96.10% | 0.20% | 100.00% | 25.30% | 90.30% |
|  | (14.2% - 24.3%) | (95.0% - 97.1%) | (0.1% - 0.3%) | (100.0% - 100.0%) | (24.3% - 26.3%) | (89.9% - 90.7%) |
| RTI | 68.60% | 99.80% | 89.70% | 99.00% | 80.40% | 98.70% |
|  | (54.3% - 82.8%) | (99.6% - 100.0%) | (86.8% - 92.6%) | (98.8% - 99.3%) | (78.0% - 82.8%) | (98.5% - 98.9%) |
| Other injuries | 62.10% | 99.30% | 34.30% | 99.60% | 19.40% | 97.40% |
|  | (47.5% - 76.6%) | (98.9% - 99.7%) | (30.6% - 38.1%) | (99.5% - 99.7%) | (16.1% - 22.6%) | (97.3% - 97.6%) |
| Ill def | 0.00% | 100.00% | 0.00% | 100.00% | 16.30% | 98.30% |
|  | (0.0% - 0.0%) | (100.0% - 100.0%) | (0.0% - 0.0%) | (100.0% - 100.0%) | (13.8% - 18.7%) | (98.1% - 98.5%) |
| Suicide | 100.00% | 99.50% | 75.40% | 99.90% | 75.40% | 99.60% |
|  | (100.0% - 100.0%) | (99.2% - 99.8%) | (73.0% - 77.9%) | (99.8% - 99.9%) | (72.5% - 78.4%) | (99.5% - 99.7%) |
| Maternal | 20.80% | 99.70% | 0.00% | 100.00% | 3.00% | 99.50% |
|  | (7.7% - 34.0%) | (99.3% - 100.0%) | (0.0% - 0.0%) | (100.0% - 100.0%) | (1.6% - 4.5%) | (99.4% - 99.6%) |
| **OVERALL** | **44.90%** | **96.10%** | **50.30%** | **96.50%** | **50.90%** | **96.50%** |
|  | **(42.6% - 47.2%)** | **(95.9% - 96.2%)** | **(49.8% - 50.8%)** | **(96.4% - 96.5%)** | **(50.4% - 51.3%)** | **(96.5% - 96.5%)** |

Acute resp=acute respiratory; TB=pulmonary TB; Other infect.=other and unspecified infections; Nutr. & endo.=nutrition and endocrine; Cirrhosis=live cirrhosis; RTI=road and transport injuries.
